# Development of Low-Pressure Die-Cast Al–Zn–Mg–Cu Alloy Propellers Part II: Simulations for Process Optimization

**DOI:** 10.3390/ma17164027

**Published:** 2024-08-13

**Authors:** Min-Seok Kim, Soonho Won

**Affiliations:** 1 Department of Materials Science and Engineering, Hanyang University, 222, Wangsimni-ro, Seongdong-gu, Seoul 04763, Republic of Korea; 2 Materials Testing & Reliability Division, Korea Institute of Materials Science, 797, Changwon-daero, Seongsan-gu, Changwon-si 51508, Republic of Korea; wsh@kims.re.kr

**Keywords:** Al-Zn-Mg-Cu alloy, low-pressure die-casting, simulation, propeller, hot tearing

## Abstract

With the increasing demand for high-performance leisure boat propellers, this study explores the development of high-strength aluminum alloy propellers using the low-pressure die-casting (LPDC) process. In Part I of the study, we identified the optimal alloy compositions for Al-6Zn-2Mg-1.5Cu propellers and highlighted the challenges of hot tearing at the junction between the hub and blades. In this continuation, we developed a coupled thermal fluid stress analysis model using ProCAST software to optimize the LPDC process. By adjusting casting parameters such as the melt supply temperature, initial mold temperature, and curvature radius between the hub and blades, we minimized hot tearing and other casting defects. The results were validated through simulations and practical applications, showing significant improvements in the quality and structural integrity of the propellers. Non-destructive testing using X-ray CT confirmed the reduction in internal defects, demonstrating the effectiveness of the simulation-based approach for alloy design and process optimization.

## 1. Introduction

With the recent advances in the leisure boat industry, the demand for propeller products used in boat propulsion systems has increased steadily [1,2,3,4,5,6]. Leisure boat propellers are primarily made from commercially available materials such as stainless steel or casing aluminum alloys [7,8]. Stainless steel propellers are widely used for high-power applications due to their superior properties, but they are expensive due to the difficulty of the manufacturing process [8,9]. On the other hand, cast aluminum alloys are easier to manufacture due to their relatively low melting points, but they are limited to low-power applications because of their material properties [9,10]. By applying high-strength aluminum alloys, it is possible to overcome the disadvantages of these two materials, offering excellent mechanical properties and relatively reasonable prices for product use [9].

In our previous study, Part I, we aimed to develop an alloy that is suitable for the manufacture of propeller products by applying the 7xxx series high-strength aluminum alloy [10]. The initial prototype production using this high-strength aluminum alloy revealed casting defects such as incomplete mold filling and cracks due to the wide solidification temperature range, high solidification shrinkage, and thermal contraction of the alloys. Particularly, hot tearing occurred at the junction between the propeller hub and the blades, significantly degrading the product’s quality. In Part I of the study, we focused on alloy optimization as an effective method for controlling hot cracking in propellers. Constrained rod casting was performed on 7000 series commercial alloys to evaluate their susceptibility to hot tearing. Based on these data, a related thermal fluid stress simulation model for predicting hot tearing was developed. Using the model, the hot tearing susceptibility was predicted for various alloy compositions, leading to the determination of the optimal alloy composition.

In the manufacturing process of traditional propeller products, conventional gravity casting processes are predominantly used [11]. However, controlling casting defects with the conventional process proves challenging for producing high-strength aluminum alloys, making it difficult to manufacture high-quality products [12]. Among various casting processes, the low-pressure die-casting (LPDC) process is effective for producing high-quality castings and is suitable for mass-production [13]. In this process, molten metal is supplied to the mold from the bottom to the top, resulting in reduced turbulence and stable mold filling, thereby minimizing porosity [13]. In the previous Part I study, we attempted to produce prototype propellers using two selected alloys, Al-6Zn-2Mg-0.5Cu and Al-6Zn-2Mg-1.5Cu (in wt.%), with the mass-production-capable LPDC process [10]. For the Al-6Zn-2Mg-0.5Cu propeller, it was possible to manufacture a high-quality product with no visible casting defects. However, for the Al-6Zn-2Mg-1.5Cu propeller, hot tearing was still observed at the junction between the hub and the blades. Therefore, further research is deemed necessary to control these casting defects and establish stable mass-production conditions.

The LPDC process requires various process variables, including molten metal supply temperature, initial mold temperature, melt pressure, and mold material and design. Therefore, optimizing the process through trial and error by adjusting these variables on the mass-production line has its limitations. Thus, the application of simulation technology is essential for efficient process optimization.

Generally, the use of well-developed commercial software allows for convenient and practical simulations [14]. The most widely used commercial software packages for casting process simulation analysis includes ABAQUS, ProCAST, and MAGMASOFT [14,15,16,17,18]. In previous studies, Wei et al. [15] developed a thermal fluid simulation model for the LPDC process using ABAQUS software, demonstrating its ability to analyze not only the solidification behavior of the alloy but also the deformation behavior of the mold due to thermal stress. Zhang et al. [16] also utilized ABAQUS software to develop a 3D model of the LPDC process for aluminum wheel manufacturing, showing that this model could accurately predict the temperature distribution in the actual process. Ou et al. [17] used ProCAST software to develop a simulation model for the LPDC process of aluminum wheels, optimizing the model for predicting solidification behavior and casting defects using various experimental results. Dong et al. [13] employed ProCAST software to predict molten metal filling and solidification, as well as casting defects during aluminum wheel manufacturing, discussing the correlation between product defects and mechanical properties. Sun et al. [18] effectively utilized MAGMASOFT to predict gas entrainment behavior in the LPDC process for manufacturing aluminum engine crankcases. Additionally, previous research has shown that these commercial software tools can be used effectively not only for casting processes like LPDC but also for predicting hot tearing phenomena occurring during solidification [19].

Among the commercial software introduced above, ProCAST software is a highly powerful tool, as it employs the finite element method, allowing for simultaneous thermal fluid stress analysis during the casting process [20]. It also excels in analyzing casting defects such as microstructure, hot tearing, misrun, and porosity, making it exceptionally effective for casting process optimization [20,21,22]. The simulation results using the commercial software introduced above are mostly focused on the interpretation of specific phenomena. The ultimate goal of our series of studies is to demonstrate the entire process from alloy improvement to process and prototype optimization using commercial software for more effective product development. Specifically, we aim to show that even small and medium-sized enterprises with limited experimental resources can efficiently conduct product development by leveraging simulation technology and a fundamental understanding of solidification theory.

In this Part II study, we aim to develop a coupled thermal fluid stress analysis model for the LPDC process that is used in propeller manufacturing, utilizing the commercial ProCAST software. The hot tearing behavior of the high-strength Al-Zn-Mg-Cu alloy derived from the previous Part I study will be predicted. Additionally, the influence of process variables on the occurrence of hot tearing will be analyzed. Ultimately, our goal is to determine the optimal process conditions and apply them to the mass-production process to manufacture propeller prototypes with suppressed hot tearing.

## 2. Materials and Methods

### 2.1. Simulation Model of LPDC Process

#### 2.1.1. Model Geometry and Mesh

**Figure 1:**
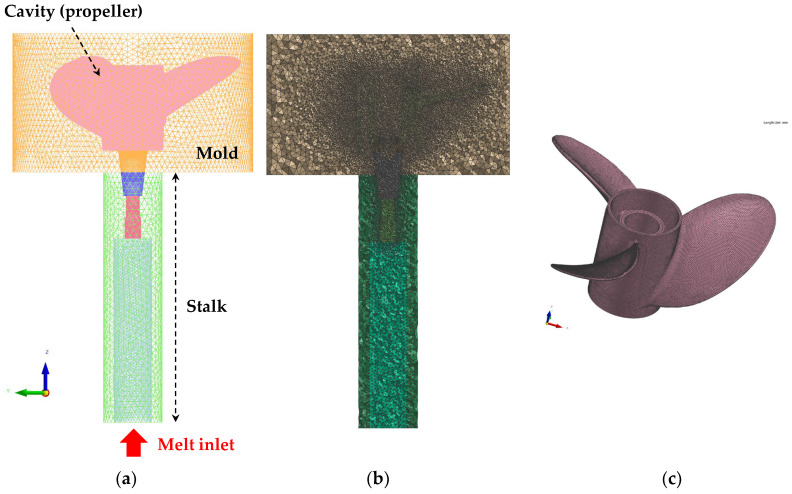
Simulation model of the propeller LPDC process: (**a**) 3D wireframe image of the 3D mesh; (**b**) vertical central cross-section mesh of the 3D solid mesh; and (**c**) mesh of the propeller part.

To manufacture high-strength aluminum propellers, simulations and prototype production were conducted using the Al-6Zn-2Mg-1.5Cu (in wt.%) alloy derived from the previous Part 1 study. Figure 1 presents a 3D simulation model of the propeller low-pressure casting process. The simulation model utilized the finite element method for thermal flow stress analysis. The model was designed considering the actual mass-production process size from the stalk part submerged in the holding furnace to the mold part. The mesh utilized tetrahedral elements, with size variations based on the importance of the analysis and an efficient calculation time. The differences in mesh size were applied using the software’s auto-mesh feature, which gradually adjusted the sizes based on the 2D mesh size at the boundaries of each part. As shown in Figure 1b, the mesh size of the propeller part, where solidification occurs, was set to approximately 3 mm, using the finest elements. The rest of the mold and stalk parts gradually increased in size from the interface with the propeller part, with the surface area set to around 10 mm.

#### 2.1.2. Casting Parameters and Boundary Conditions for LPDC Simulation

For the finite element analysis of the propeller LPDC process, the commercial software ProCAST package (ver. 2021) provided by the ESI Group was used. This software package provides optimal simulation conditions for various casting processes, including the LPDC process, with modules that are tailored to each process. Additionally, it features an advanced GUI that allows users to easily review simulation results, making it accessible for novice researchers to adapt quickly and easily. The material properties of each element in the simulation model were calculated using the database provided by the PanAl2021 software included with ProCAST. In the previous Part I study, it was demonstrated that when calculating the material properties under back diffusion cooling conditions, the eutectic phase fraction was more accurately predicted compared to the Scheil condition when compared with experimental results [23,24]. In this study, as in the previous study, the material properties were calculated considering the back diffusion cooling conditions [10,25]. The thermo-physical properties of the aluminum, mold, and stalk parts used in this study are summarized in the previous Part I study [10].

The initial and boundary conditions for the LPDC process simulation are summarized in Table 1. To predict the tendency of hot tearing occurrence under various casting conditions, simulations were conducted with different melt supply temperatures and initial mold temperature conditions. The values in Table 1 represent the standard conditions used in the mass-production process, while the ranges in parentheses indicate the range of conditions that were controlled in this study. The initial stalk temperature was set to the same value as the melt supply temperature for each condition, considering the stalk being submerged in the molten metal in the holding furnace.

**Figure 2:**
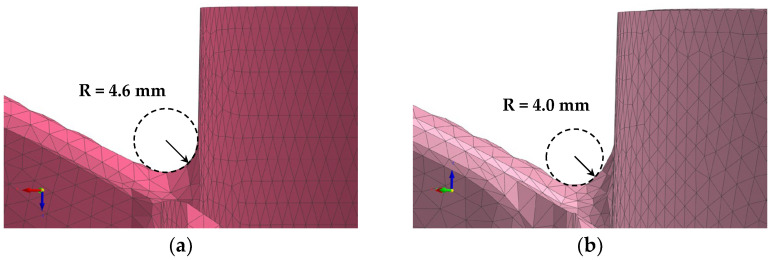
Curvature radius conditions between the hub and blades of the propeller: (**a**) 4.6 mm; (**b**) 4.0 mm.

In addition to standard casting conditions, the angle between the hub and blades of the propeller was adjusted through mold processing to control hot tearing. Figure 2 shows the propeller blade connection before and after mold modification. Before the modification, the curvature radius was approximately 4.6 mm, while after the mold improvement, the curvature radius was approximately 4.0 mm in the manufactured propeller product.

To reflect mold preheating and repetitive production lines in the LPDC mass-production setting, mold preheating and cycle analysis were conducted. In the LPDC simulation calculations, conducting preliminary thermal simulations for mold preheating and 10 test castings were performed to account for the initial mold temperature distribution under actual operational conditions. The preliminary simulation conditions are summarized in Table 2.

#### 2.1.3. Simulation Model for Predicting Hot Tearing

To investigate the susceptibility to hot tearing in LPDC propellers, a coupled thermal flow stress analysis was conducted. For stress analysis, the effective plastic strain (EPS) model supported by ProCAST software was utilized. The EPS model can be defined as the total accumulated plastic strain occurring during the solidification of each part. Therefore, EFS, $\bar{\varepsilon }$, can be expressed by the following equation [26]:

(1) $$\bar{\varepsilon }=\int_{0}^{t}\sqrt{\frac{2}{3}{\dot{\varepsilon }}^{P}:{\dot{\varepsilon }}^{P}}d\tau ,\;t_{C}\;\leq t\;\leq \;t_{S}$$

where ${\dot{\varepsilon }}^{P}$ is the effective plastic strain rate. Generally, the onset of significant stress during solidification is related to the solid fraction where interaction between dendrite branches begins, known as the dendrite coherency point [27]. Therefore, considering the dendrite coherency point of grain-refined aluminum alloys, stress analysis was conducted starting from a solid fraction of 0.5 or higher.

### 2.2. Defect Analysis Method for LPDC Propellers

The surface microstructure of hot tearing in the propeller was observed using a scanning electron microscope, SEM JEOL JSM-6610LV (Tokyo, Japan). To analyze the overall internal defects of the propeller, a non-destructive testing method, X-ray CT, was used. The specific specifications of the equipment are well documented elsewhere [28].

## 3. Results and Discussion

### 3.1. Hot Tearing in LPDC Propellers

**Figure 3:**
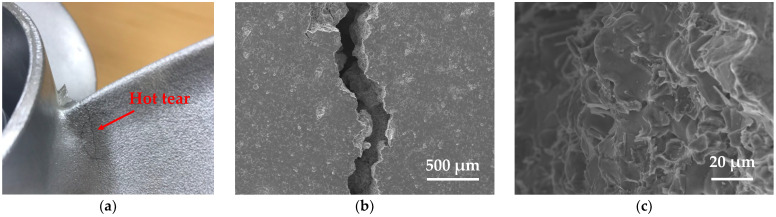
Hot tearing in Al-6Zn-2Mg-1.5Cu propeller: (**a**) appearance of hot tearing; (**b**) SEM image of hot tearing; and (**c**) SEM image of fracture surface microstructure of hot tearing.

In propeller products, there is typically a junction between the relatively thick hub and the thin blades. This area features a sharply bent shape, which can easily lead to stress concentration due to solidification shrinkage or thermal contraction during casting. Figure 3 shows the appearance of cracks and SEM images of an Al-6Zn-2Mg-1.5Cu alloy propeller manufactured using the LPDC process. These fine cracks primarily occurred at the junction between the hub and the blades of the propeller. The cross-sectional microstructure of the crack surface revealed smooth surfaces of dendrites or grains, indicating that these cracks were primarily caused by hot tearing during the solidification process. This hot tearing is a critical defect that is irreparable after casting. This study, therefore, utilizes process optimization through simulation techniques to mitigate the occurrence of such hot tearing.

### 3.2. Influence of Casting Conditions on Hot Tearing in LPDC Propeller Simulation

**Figure 4:**
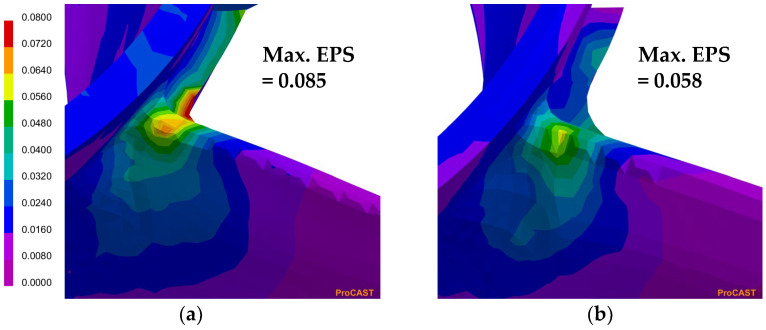
Simulation results of EPS prediction according to the curvature radius between the blade and hub of the propeller: (**a**) R = 4.6 mm; (**b**) R = 4.0 mm.

In order to effectively control hot tearing in the LPDC process, a simulation model was used to investigate the effects of various process conditions on the occurrence of hot tearing. Figure 4 shows the EPS prediction results according to the curvature radius at the junction of the hub and blade in the LPDC propeller. The simulation results indicate that the EPS prediction, which highlights the areas where deformation is concentrated during solidification, accurately predicts the regions where hot tearing occurs in the actual propeller product. The simulation, conducted by varying the curvature radius, showed that as the radius was changed from 4.6 mm to 4.0 mm, the maximum EPS value significantly decreased from 0.085 to 0.058. The simulation results shown in Figure 2 and Figure 4 indicate that by adding material to the junction area to create a relatively smoother transition structure, the concentration of deformation can be dispersed. This approach is highly effective in significantly reducing the susceptibility to hot tearing.

**Figure 5:**
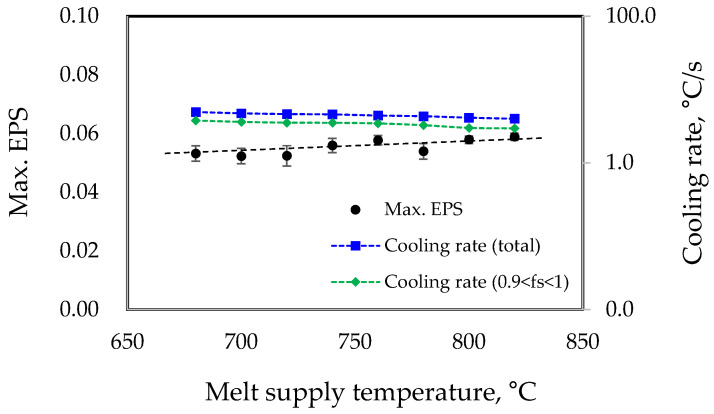
Simulation results on the effect of melt supply temperature on EPS and cooling rate in the Max. EPS region.

To investigate the influence of casting conditions on the occurrence of hot tearing, simulations were conducted using a propeller model with an improved curvature radius (R = 4.0 mm). In the LPDC process, the melt supply temperature is the casting condition that can most easily be adjusted. In this study, simulations were performed with melt supply temperatures ranging from 680 °C to 820 °C, considering the maximum feasible range in the mass-production line. Figure 5 illustrates the variation in the predicted Max. EPS values and the cooling rate in the Max. EPS region according to different molten metal supply temperature conditions. For the cooling rate, two values were calculated: the total value representing the average cooling rate from the liquidus to the solidus temperature, and the cooling rate within the solid fraction range of 0.9 < f_s_ < 1, where hot tearing predominantly occurs [29]. As shown in the figure, the EPS appears to increase slightly with the increase in the molten metal supply temperature. However, within the given range of conditions, considering the difference between the maximum value of 0.059 and the minimum value of 0.052, no significant effect was observed.

**Figure 6:**
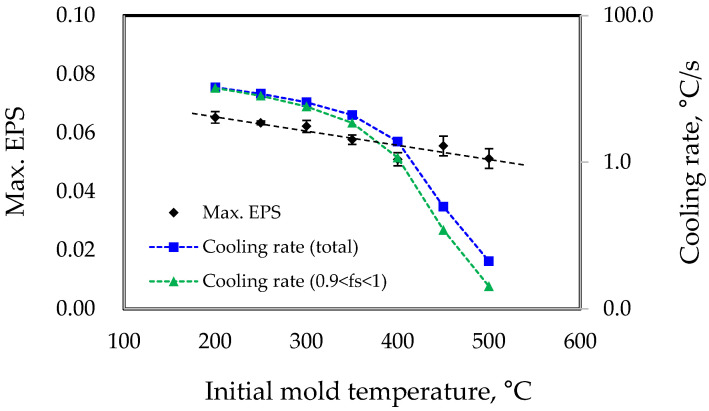
Simulation results on the effect of initial mold temperature on EPS and cooling rate in the Max. EPS region.

In the LPDC process, the initial mold temperature is also a critical factor influencing casting defects. This study conducted simulations to investigate the changes in EPS values by varying the initial mold temperature widely, from 200 °C to 500 °C. It is important to note that excessively low initial mold temperatures can lead to defects such as incomplete filling of the molten metal, while excessively high temperatures can cause secondary defects such as decreased mechanical properties of products due to lower cooling rates. In this study, conditions were controlled solely to understand the tendencies of hot tearing. Figure 6 shows the changes in Max. EPS and the cooling rate in that region according to the initial mold temperature. The simulation results indicate that as the initial mold temperature increases, the EPS value decreases from 0.065 to 0.051. Therefore, it can be concluded that increasing the mold temperature helps to suppress hot tearing.

In addition to geometrical conditions such as the curvature radius between the propeller hub and blades, parameters like the melt supply temperature and initial mold temperature are closely related to the cooling rate during casting. As previous studies have shown, the faster the cooling rate is, the higher the deformation rate in the final solidification region during casting is [29]. This reduces the time available for the residual liquid to heal the gaps formed between the grains due to solidification shrinkage or thermal contraction, ultimately increasing the susceptibility to hot tearing. Of course, as the amount of eutectic liquid in the final solidification zone decreases, the hot tearing susceptibility can increase, and this amount can also be influenced by the cooling rate [29,30,31]. However, in this study, the solid fraction distribution with respect to temperature was calculated under the same cooling conditions for a specific alloy, and thus, this factor can be somewhat disregarded. Therefore, the variation in EPS values according to initial casting conditions is primarily attributed to changes in the cooling rate. The simulation results shown in Figure 6 indicate that as the initial mold temperature varies from 200 °C to 500 °C, the average cooling rate decreases from approximately 10.6 °C/s to 0.04 °C/s. This implies that a reduction in the cooling rate by more than two orders of magnitude is necessary to achieve a significant hot tearing suppression effect. Generally, for the same alloy, as the cooling rate decreases, the amount of eutectic liquid in the final solidification zone tends to increase [31]. Therefore, in actual casting processes, as the initial mold temperature increases, and hence the cooling rate significantly decreases, the effect of reducing hot tearing susceptibility can be more pronounced. However, it should also be noted that excessively low cooling rates can lead to the coarsening of the microstructure and an increase in segregation, which can degrade the mechanical properties [32].

In the calculations above, the initial mold temperature conditions were set to a uniform temperature. However, in actual mass-production settings, torches are often used to preheat the inner cavity of the mold for operational convenience. In such cases, even if the cavity surface reaches the desired temperature, there can be a temperature deviation of over 100 °C between the cavity surface and the outer surface of the mold. Additionally, in mass-production processes, the repeated production cycles can cause temperature deviations between the inner wall and the outer surface of the mold. Therefore, to consider mass-production conditions, this study conducted initial preheating and cycle analyses under the conditions specified in Table 2 to determine the initial mold temperature conditions.

**Figure 7:**
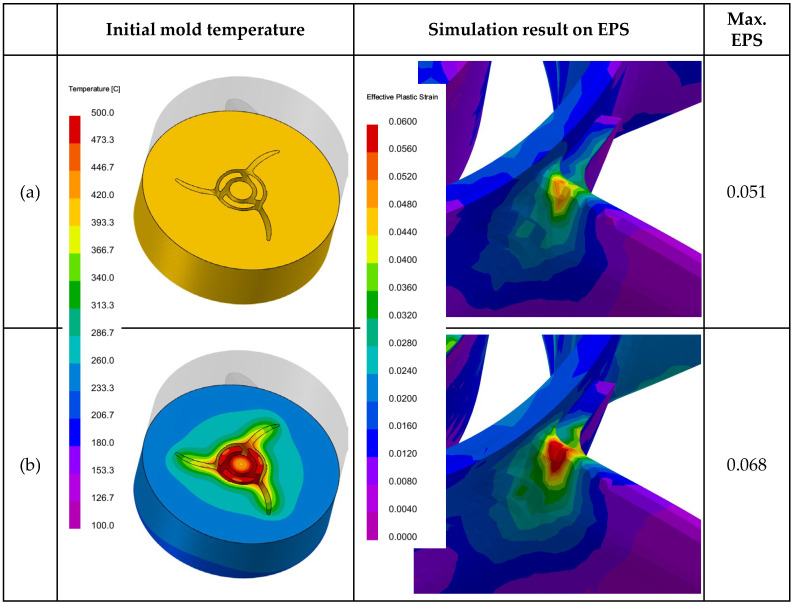
Initial mold temperature conditions and the resultant EPS predictions: (**a**) uniform initial mold temperature condition of 400 °C; (**b**) cycle-analyzed initial mold temperature condition.

Figure 7 shows the initial temperature distribution conditions of the molds and the predicted EPS results under the simulation conditions of a melt supply temperature of 760 °C and an initial mold temperature of 400 °C. The cycle-analyzed initial mold temperature condition used was approximately 400 °C near the area where hot tearing occurs. As shown in the figure, despite similar temperatures inside the mold, the EPS values changed significantly depending on the overall temperature distribution of the mold. This is because, compared to uniform initial mold temperature conditions, a greater temperature difference between the interior and exterior of the mold can lead to undesired higher cooling rates. When the average cooling rates near the hot tearing region were calculated for the results in Figure 7, the uniform temperature condition and the non-uniform temperature condition showed cooling rates of approximately 1.9 °C/s and 4.4 °C/s, respectively. Therefore, when applying simulation results to practical mass-production lines, these factors must be carefully considered. Additionally, these results suggest that maintaining a uniform mold temperature by inserting internal heating elements can effectively control hot tearing, even under the same conditions.

### 3.3. Quality of Developed Al-6Zn-2Mg-1.5Cu Propellers

**Figure 8:**
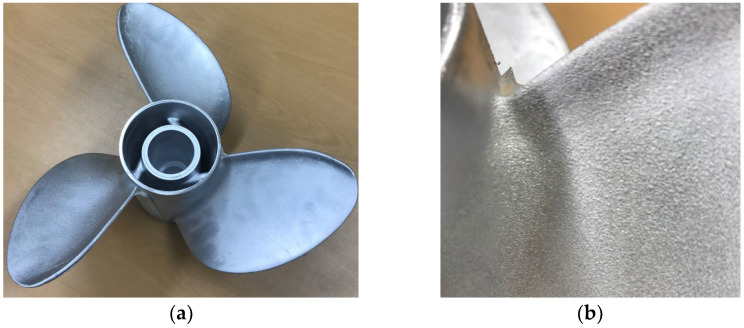
Developed Al-6Zn-2Mg-1.5Cu alloy propeller: (**a**) propeller appearance; (**b**) appearance of the hub and blade connection of the propeller.

Based on the simulation results, the optimal process conditions were applied to the LPDC mass-production line to manufacture prototype propellers. Figure 8 shows the appearance of the Al-6Zn-2Mg-1.5Cu alloy propeller fabricated under the improved conditions, as well as a close-up of the hub and blade connection where hot tearing frequently occurred. It was confirmed that high-quality propeller products could be manufactured without hot tearing under the optimized alloy and process conditions.

**Figure 9:**
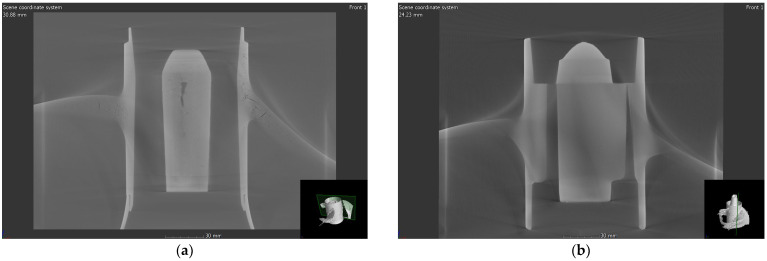
Internal defect investigation of aluminum alloy propellers through CT analysis: (**a**) commercially available Al-7Si-0.3Mg alloy propeller; (**b**) developed LPDC Al-6Zn-2Mg-1.5Cu propeller.

In addition to externally observed hot tearing, non-destructive testing using X-ray CT was conducted to investigate the internal defect distribution in the developed propeller products. Figure 9 presents the CT analysis results of commercially available Al-7Si-0.3Mg cast propellers and the Al-6Zn-2Mg-1.5Cu propeller products developed in this study. Despite being a traditional casting alloy, the alloy in Figure 9a exhibits a large distribution of internal defects such as shrinkage cavities and pores. In contrast, the developed product (Figure 9b) demonstrates the ability to produce high-quality propellers with no significant internal defects. These results indicate that the simulation-based approach for alloy design and process optimization, as demonstrated in Parts I and II of this study, is highly practical and effective. While comprehensive data collection through extensive experiments is ideal for research and development, this study aimed to show that even small and medium-sized enterprises with limited R&D resources can achieve efficient product development using simulation technology. It is hoped that the findings of this study will not only contribute to the expansion of the high-strength propeller market but also encourage the development of various high-quality cast products through practical research methods that are similar to those employed in this study. Furthermore, integrating these simulation techniques with the recently popularized machine learning technology in the future could enable even more effective research and development.

## 4. Conclusions

The LPDC process conditions for the high-strength Al-6Zn-2Mg-1.5Cu alloy were successfully optimized, effectively addressing hot tearing issues at the junction between the hub and blades in propellers. Through simulation-based process optimization using ProCAST software, the optimal casting parameters, including melt supply temperature and initial mold temperature, were identified. Specifically, simulations demonstrated that increasing the initial mold temperature from 200 °C to 500 °C significantly reduced the maximum effective plastic strain (EPS) from 0.065 to 0.051. Additionally, optimizing the curvature radius between the hub and blades from 4.6 mm to 4.0 mm reduced the maximum EPS value from 0.085 to 0.058. Practical applications in the mass-production line confirmed the feasibility of producing high-quality propellers with minimal defects under these optimized conditions. X-ray CT analysis validated the substantial reduction in internal defects, showing a significant improvement compared to commercially available propellers. These results highlight the practicality and efficiency of using simulation technology for product development, with the optimized propellers exhibiting no significant internal defects and demonstrating improved structural integrity.

## Figures and Tables

**Table 1:** The initial and boundary conditions for LPDC simulation.

Condition	Value
Mold/Stalk materials	H13/Al_2_O_3_
Melt supply temperature	760 °C (680~820 °C)
Initial mold temperature	350 °C (200~500 °C)
Initial stalk temperature	760 °C (680~820 °C)
Ambient temperature	20 °C
Inlet pressure	Pressurization: 0→0.3 bar for 8 s Holding: 0.3 bar for 30 s Melt drain: 38 s
Mold/Casting heat transfer coefficient	1400 W/m^2^K (Ref. [17])
Mold/Air heat transfer coefficient	20 W/m^2^K (Ref. [17])

**Table 2:** Pre-simulation conditions for mold preheating and cycle analysis.

Mold Heating Stage	Condition	Value
Mold preheating	Initial mold temperature	100 °C
	Mold/Torch flame heat transfer coefficient	50 W/m^2^K
	Heating time	1200 s
Cycle analysis	Number of cycles	10
	Mold opening time	180 s
	Part ejection time	210 s
	Time of the die spraying	300 s
	Mold closing time	420 s
	Mold/spray coefficient	300 W/m^2^K
	Spray temperature	20 °C

## Data Availability

The raw data supporting the conclusions of this article will be made available by the authors on request.
